# Attachment Styles and Communication of Displeasing Truths

**DOI:** 10.3389/fpsyg.2020.01065

**Published:** 2020-06-05

**Authors:** Isora Sessa, Francesca D’Errico, Isabella Poggi, Giovanna Leone

**Affiliations:** ^1^Department of Psychology, Sapienza University of Rome, Rome, Italy; ^2^Fil.Co.Spe. Department, Roma Tre University, Rome, Italy; ^3^Coris. Department, Sapienza University of Rome, Rome, Italy

**Keywords:** attachment styles, truthful communication, displeasing truth, frankness, mitigation, politeness

## Abstract

This work explores how humans manage the communication of a displeasing and face-threatening truth and how the communicative strategies of the sender and the reaction of the receiver are influenced by their attachment style. Two experimental studies demonstrate that the attachment styles of both senders and receivers can influence the communicative styles of the sender, the emotions that both senders and receivers feel or attribute to their interlocutor, and the reactions of the receivers. In Study 1, couples of participants played a bogus computer game, ostensibly to test their abilities. Subsequently, “the spokesperson” was given the task to communicate to the “the receiver” a bogus low score of the other and a high score of oneself. Finally, all participants completed an adult Attachment Style Questionnaire (ASQ). A content analysis of the verbal messages of the spokespersons showed two main communication styles: frankness and mitigation. The results suggest that the spokespersons’ attachment style influences these communication styles. Using a similar procedure, Study 2 showed that spokespersons with a high avoidant attachment more frequently used frankness when communicating low scores to the receivers. Furthermore, the emotions and impressions reported by anxious and avoidant spokespersons and receivers, respectively, confirm the negative model of the self or the other, typical, respectively, in anxious and avoidant attachment. The detection of communicative strategies stemming from different attachment styles might be of use in user modeling and the planning of personalized systems.

## Introduction

Acquiring beliefs about the external world and themselves is a primary need for humans to achieve their goals; this is why communication – and specifically, telling the truth – is generally considered as an act of cooperation (Grice, 1975) and reciprocal altruism (Castelfranchi and Poggi, 1998), whereas deceiving, i.e., providing false or withholding true information, is viewed as a sin by religions and a harmful and morally execrable action by ethics (Bok, 1978; Augustinus, 1994; Kant, 1996). If pragmatics (Grice, 1975; Sperber and Wilson, 1995; Castelfranchi and Poggi, 1998) considers telling the truth as the main principle of communication, according to psychological studies, most people believe that this is the norm in most human interactions (Moghaddam, 2002; Levine, 2014): they expect to be believed and at the same time do not doubt the veracity of the information received (Kalbfleisch and Docan-Morgan, 2019); this facilitates social interaction and the understanding of others (Kalbfleisch and Docan-Morgan, 2019), producing indisputable beneficial effects, in terms of trust, well-being, and security.

Yet, although true beliefs are generally of help for people, sometimes they may hurt, since they may cause painful emotions, from fear to worry to anxiety, from disappointment to guilt to shame.

Therefore, people often refrain from telling displeasing truths to others (Moreno et al., 2016; Levine and Cohen, 2018); although sincerity is a feature of the utmost importance for interpersonal judgment (Anderson, 1968; Goodwin et al., 2014), it can also be viewed as an act of cruelty (Tagliapietra, 2003), while with holding unpleasant information is seen as a way to protect the other from disrupting emotions.

When a sincere answer might be unpleasant for the other or themselves (Levine and Cohen, 2018), people must decide whether and how to communicate negative news or criticism (Stone et al., 2010), and due to anxiety or social unease (Molinsky and Margolis, 2005; Margolis and Molinsky, 2008), they may not be sincere, rather simply being pleasant to build quiet social relationships (Rosen and Tesser, 1970; Tesser et al., 1971; Lee, 1993). This is the bulk of “white lies,” which sometimes stem out of selfish aims but are often motivated by altruistic and pro-social goals (Castelfranchi and Poggi, 1998; Erat and Gneezy, 2012; Biziou-van-Pol et al., 2015).

Within the displeasing beliefs one may decide to withhold from an interlocutor, two main types can be distinguished, according to the negative emotions they may induce: it is not the same to conceal to a patient she has a terminal cancer and to tell a writer her novel cannot be published because it is boring. The former may induce stress or terror; the latter challenges the very image of the person. Among “white lies,” we can count both disappointing, scaring, or worrying news of the former type and face-threatening ones; within these last is politeness, viewed in the psychological literature (Axia, 1999) as the ability to predict and prevent any possible discomfort of the other, protecting his/her need to be free and autonomous and his/her self-esteem and emotions, and by pragmatic studies (Lakoff, 1973; Brown and Levinson, 1978; Leech, 1983) as the set of linguistic strategies aimed at saving the interlocutor’s “face” (Goffman, 1963, 1981), the image that individuals show of themselves during interactions with others. Two strategies to maintain comfortable interactions, while not directly providing true information, are *equivocation* and *avoidance* (Kalbfleisch and Docan-Morgan, 2019), i.e., providing information that can be interpreted in various ways or shifting to other topics. In other cases, one does not tell the exact truth, because one thinks the other does not really want to know it.

In sum, conveying a displeasing truth to others may trigger unpleasant emotions in the sender as much as in the receiver (Poggi and D’Errico, 2010, 2018). Here it is relevant whether the sender is empathic toward the receiver, and this is mediated by his/her attitude toward and relationship with the other, which may depend in turn on some individual characteristics of the sender, including his/her attachment style. This article presents two studies aimed at exploring the relationships between attachment styles of senders and receivers and their ways to tell and to react to a displeasing truth.

Attachment theory conceptualizes “the propensity of human beings to make strong affectional bonds to particular others” (Bowlby, 1977, p. 201). The attachment system develops in childhood: infants seek proximity with their caregivers, especially in conditions of danger or threat. Children, over time, internalize their early attachment relationships, and their experience with caregivers finally forms a prototype (*internal working model of attachment*) for adult relationships (Bowlby, 1973, 1982; Holmes, 1993; Meyer and Pilkonis, 2001) that remains active throughout the life span (Bowlby, 1977). Three main adult attachment styles have been identified (e.g., Hazan and Shaver, 1987; Kobak and Sceery, 1988; West and Sheldon, 1988):

1. Secure attachment that represents a positive model of the self and security of relationships (Mikulincer and Shaver, 2016). Secure individuals have a sense of worthiness (lovability) plus an expectation that other people are generally accepting and responsive;
2. Anxious attachment that represents a negative model of the self and relational anxiety (Bartholomew and Horowitz, 1991). Anxious individuals have a sense of unworthiness (unlovability) combined with a positive evaluation of others and a need for acceptance of valued others;
3. Avoidant attachment that represents a negative model of the other and avoidance of relationships (Bartholomew and Horowitz, 1991). Avoidant individuals have a sense of love-worthiness combined with a negative disposition toward other people and a need to protect themselves against disappointment by avoiding close relationships and maintaining a sense of independence and invulnerability.

Since different attachment styles – secure, anxious, and avoidant – result in different relationships with the other, this work investigates if different attachment styles affect the ways in which people convey a displeasing truth, more specifically evaluative information concerning the receiver that may hurt his/her face and hence trigger emotions of shame, humiliation, and embarrassment.

Actually, to the best of our knowledge, there is no specific research devoted to this topic. Some studies investigate how attachment relates to the self-assessed habit of lying (Cole, 2001; Ennis et al., 2008; Gillath et al., 2010), but they do not directly observe the actual deceptive communication of people with secure or insecure attachment styles. Moreover, from a methodological point of view, self-report measures are commonly used to study deception, but the use of such self-assessed evaluations has been criticized because, for reasons of social desirability, participants’ self-assessments may be distorted (Elaad et al., 2012); to overcome the limitations of self-report measures, a new methodological approach is needed (Leone et al., 2016; Migliorisi, 2019). The goal of our research is therefore to assess the relationship between attachment style and the communication of a displeasing truth, by observing how people with secure or insecure attachment styles actually cope with the experimental task of telling negative evaluative information to a receiver they did not formerly know.

In the following, we present two studies investigating this topic.

## Study 1

### Aims

The goal of the first study was to observe how people communicate some face-threatening news to others and to establish whether their style of communicating such displeasing truth is in some way related to their attachment style.

### Procedure

Considering that traditional self-report methods do not allow a reliable assessment of people’s sincerity (DePaulo et al., 2003; Sporer and Schwandt, 2007), a novel and quite complex procedure has been put in place in order to observe people in a real situation in which (1) they had to decide how much and how to tell when telling another a face-threatening truth and (2) there was also the chance to video-record and analyze real interactions. To carry out such procedure, we recruited 68 participants among undergraduate students of Social Psychology and coupled them into 34 pairs. A *cover story* was used asking them to participate in marketing research on the consumption of cultural products among young people, proposed by a marketing company independent from the university. The two participants in each pair (previously unknown to each other) were invited through an email message from the fictitious marketing company informing them that the research comprised playing a game in pairs and that the winning pair would receive a €200 voucher to spend in a store with media products (books, music CDs, video movies, and TV series). It was specified that the other pairs would also receive €20 voucher to spend in the same store. The participants were told that the research consisted of two phases. First, they would go to the department where the research should have taken place and meet with an unknown participant with whom the experimenter had paired them. Second, a few days later and during class, they would complete a short questionnaire and receive information about the study.

### Phase I

During the first phase, both members of the pair were informed that before competing against the other pairs, each member would individually play a computer game to test his/her previous skills. They were also told that based on the results of this test, the pair could decide whether to participate in the playful competition with the other pairs. Furthermore, the participants were told that in the second phase of the procedure, they would complete a questionnaire (the ASQ, Attachment Style Questionnaire). Subsequently, they were asked to sign an informed consent form. This first consent form, right for research purposes, did not communicate that some participants would be videotaped during the first phase.

### Setting

The pair was invited to sit in a room with two chairs, one in front of the other, separated by a table with only a computer set in front of one of the chairs. A hidden camera was placed in front of it on a piece of furniture. The pair would meet in this room before and after individually playing the computer game. After signing the consent form, each member of the pair was assigned a numeric code, and the experimenter told them to write it down on the questionnaire to be completed during the second phase of the procedure. Actually, this was a ploy to allow the experimenter to match the video recordings grabbed in the first phase of the procedure with the questionnaires to be administered in the second phase.

In each pair, one member, always the participant who randomly sat in the direction of the hidden camera, was chosen by the experimenter as a spokesperson. The spokesperson always received a code with an odd number, so it was possible to discriminate, among all participants, those who had played the role of spokesperson. As anticipated in the consent form, the members of the pair were reminded that they would perform a computer task in different rooms to assess their own individual skills before competition. The participants were told that a central computer would monitor their game actions in real time and process their results immediately after the test. Starting from these results, the pair could decide whether to participate together against the other pairs. The researcher specified that the pairs who chose not to participate in the game with the other pairs could still complete the questionnaire (ASQ scale) during the second part of the procedure. Finally, the researcher told the two members that she would communicate both scores only to the spokesperson and that the spokesperson would communicate the received score to the other participant. After listening to these instructions, the spokesperson was left alone in the room while the other participant was accompanied to another room. The two participants individually played the computer game in different rooms. After 7 min, the two participants were interrupted. The experimenter told the spokesperson that the average score obtained in the execution of the test was 6.2 (on a scale of 0–10). The spokesperson was informed that he/she had a high score (i.e., 8.4), while his/her partner had a very low one (i.e., 3.6). Furthermore, the experimenter pointed out to the spokesperson that the difference between the individual skills assessed by the difference between these two (bogus) scores would have penalized the pair in the competition with other pairs. The researcher also reminded the spokesperson to communicate the outcome of the test to his/her partner. For this reason, the other participant was accompanied to the room where the spokesperson was waiting for him/her. The pair had 3 min to decide whether to participate in the game against the other pairs. In this way, the spokesperson was obliged to communicate the very low score to the other participant. This bogus assessment of the individual skill that the spokesperson was expected to communicate to the other participant was the way in which a displeasing truth was introduced the procedure. Due to this methodological choice, when communicating to the other member of their couple the difference between their two bogus scores participants acted as if the content of their communication was true. This is a kind of procedure that, transiently resembling to the scenario of a game simulation, may be particularly apt for observing difficult interpersonal interactions (Leone, 2013). After 3 min, the participants had to communicate their decision, and they were reminded of the appointment for the second phase of the procedure. Therefore, in this procedure, the unpleasant truth was a low score obtained in a computer game. This truth was made particularly unpleasant to communicate and to receive because it encouraged a social comparison between the two participants. In fact, one of the two participants had not only a lower score than the average but also a lower score than his/her partner.

### Phase II

After a few days, during a class, the ASQ by Feeney et al. (1994) was administered to the participants. It asked the participants to rate (on a six-point scale) the extent to which each item described their feelings and behavior in interpersonal relationships (not necessarily romantic). Subsequently, the participants were subjected to debriefing, where the true research purposes were revealed. The researcher provided a detailed explanation of the procedure, including the fact that the playful competition would not have occurred. Finally, the students were told they had been videotaped during the first phase of the research, and in accordance with the ethical requirements of research on studies requiring a cover story, they signed a second consent form for the video recordings. They were assured that in case of non-authorization, they would receive the promised reward for their participation and that the video recordings would be irreversibly destroyed.

### Participants

Thirty-four pairs of undergraduate students participated in the research. This first study focused on the observation of the spokesperson’s communication. Therefore, the results of this first study refer only to the 34 participants (24 women and 10 men, mean age of 20) who played the role of the spokesperson.

### Analysis

#### The Communication of the Displeasing Truth

In this study, the unpleasant truth that the spokesperson had to communicate to the other participant consisted of a low score obtained in a computer game. This score was lower than both the average and also the score obtained by the spokesperson. The parts of the video recordings in which the spokespersons – who authorized use of the video recording – communicated the unpleasant truth to the other participant were selected and transcribed. In order to identify the communication styles of the spokespersons, a qualitative analysis of their verbal content was performed.

#### The Spokespersons’ Communication Styles

The corpus of interactions between spokespersons and their partners is of 4,974 words, for a total of 102 min. A qualitative analysis was carried out of the verbal content of the interaction by two judges independently, achieving a good level of agreement (*k* = 0.88), from which two main communication styles of the spokespersons emerged: *straight* and *mitigated*. By relying on previous definitions by Caffi (1999), we define mitigation as any linguistic and pragmatic strategy used by the sender of a communicative act aimed at attenuating the potential negative emotions caused to the addressee by that communicative act. In pragmatic and linguistic literature, typical examples of mitigation are indirect acts and justification moves, passive and impersonal constructions, modal adverbs, and parenthetical forms (Caffi, 2007), but the mitigation forms we consider here are general discourse strategies aimed at attenuating the addressee’s displeasure for the displeasing truth conveyed.

The spokespersons who chose a *straight* communication style communicated the unpleasant truth to the other participant without adding anything else. By contrast, the spokespersons who chose a mitigated communication style conveyed the unpleasant truth to the other participant while adding other statements, among which we can distinguish five subtypes:

– Reassuring the partner (for example, “*don’t worry*”);
– Emphasizing the difficulty of the game (“*the game wasn’t easy*”; “*the last items were very difficult*”);
– Showing surprise at one’s own score and/or underestimating one’s own capacities (“*even though I have no logical skills, strangely*.”; “*I didn’t even finish the game*”);
– Attributing to the researcher some words that she had not actually said (“*the researcher told me that our average score is 6.2*”);
– Consulting with the partner on the game (“*how would you have responded this item?*”).

#### The Spokespersons’ Attachment Style

The ASQ is a self-report and dimensional measurement of adult attachment. The ASQ was chosen because this kind of self-report measure is recommended and has adequate reliability and very good face and discriminant validity, when attachment is not a primary area of investigation (Ravitz et al., 2010). Furthermore, a dimensional questionnaire was chosen because this kind of measures does not assign individuals to categories of attachment style, but it assesses the degree to which various dimensions of attachment are present. In fact, categorical measures of attachment have been criticized theoretically, for assuming that differences among people within a category are “unimportant or do not exist” (Mikulincer and Shaver, 2007, p. 85), and analytically, for their limited statistical power compared with dimensional measures (Fraley and Shaver, 2000).

The 40 items on the ASQ include five subscales:

1. Confidence in self and others (for example, “*overall, I am a worthwhile person*”; “*I feel confident that other people will be there for me when I need them*”);
2. Discomfort with closeness (“*while I want to get close to others, I feel uneasy about it*”);
3. Need for approval and confirmation by others (“*I find it hard to make a decision unless I know what other people think*”);
4. Concern about relationships (“*I worry that others won’t care about me as much as I care about them*”);
5. Viewing relationships as secondary to achievement in various domains, such as school or career (“*achieving things is more important than building relationships*”).

In the above subscales, no. 2, discomfort with closeness, and no. 5, viewing relationships as secondary, are clearly conceptually related to avoidant attachment (Bartholomew, 1990; Collins and Read, 1990; Brennan et al., 1998; Mikulincer and Shaver, 2003, 2007). No. 4, concern about relationships, and no. 3, need for approval and confirmation by others, are conceptually related to anxious attachment (Hazan and Shaver, 1987; Bartholomew, 1990; Bartholomew and Horowitz, 1991). No. 1, confidence (in self and others), is related to secure attachment (Mikulincer and Shaver, 2007). In addition to yielding the above five scores, ASQ items can be used to form scores for propensity of attachment anxiety and avoidant attachment. In this study, the median value within each subscale was calculated, and the participants were classified based on their scores being above or below this median value. Therefore, dichotomous variables were obtained for each dimension measured by the ASQ.

### Results

The content analysis of the spokespersons’ verbal communication showed that they chose different communication styles to convey an unpleasant truth. In the 34 spokespersons, 16 chose a straight communication style (47%), while 18 utilized a mitigated communication style (53%). Therefore, we investigated whether there was a relationship between these communication styles and the spokespersons’ attachment styles. The chi-square test indicated that, among the spokespersons with “high need for approval and confirmation by others” (median value = 3) sub-group (*n* = 16), 75% of them (*n* = 12) chose the mitigated communication style, while only 25% chose a straight communication style (χ^2^ = 5.903; *p* = 0.02).

### Discussion and Conclusion

This first exploratory study produced interesting results. First, from the analysis of the verbal content expressed by the spokespersons, two different communication styles emerged: straight and mitigated. Furthermore, there was a relationship between the mitigated communication style and the dichotomous variable “high need for approval and confirmation by others.”

The need for approval and confirmation by others reflects anxious attachment that represents a negative model of the self (Bartholomew and Horowitz, 1991) and includes concerns about intimacy, jealousy, and fear of abandonment, as well as a dependency on a close other’s approval rather than an internal sense of self-worth (Brennan et al., 1998; Cole, 2001). Therefore, one might assume that people with these characteristics have more difficulty communicating an unpleasant truth and that they choose a mitigated communication style to reduce their relational anxiety. In fact, the spokespersons who chose a mitigated communication style reassured the other participant, emphasized the difficulty of the game, showed surprise at their own score, and underestimated their own capacities. Further, they attributed to the researcher some words that she had not said, and they consulted with the other participant on the game. Consistent with the definition of mitigation (Caffi, 2007), by adopting these strategies, the spokespersons modulated their communication in the direction of a mitigation to avoid potentially unpleaseant perlocutionary outcomes. Interestingly enough, this communication strategy resembles to over-helping strategies (Leone, 2012), i.e., interactions when helpers, because of their high level of anxiety due to their perception of the recipients’ vulnerability, give them a kind of help exceeding their actual needs. Similar results were shown, for instance, when mothers interacted in a game simulation with their chronically ill children (D’Errico and Leone, 2006), or when teachers interacted in a similar game simulation with pupils of immigrant families (D’Errico et al., 2010).

The results of this first study are encouraging because they suggest that the spokespersons’ attachment style influences the communication style they adopt when conveying a displeasing truth. However, this study only focused on the spokesperson’s communication: the reactions of the participants who received a displeasing truth were not observed. In any case, this study also aimed to test a new procedure, never previously used; since this procedure proved to be effective, a second more articulated study was carried out, to consider also the participants who received the displeasing truth and to provide a more fine-grained analysis of the communication of both the spokesperson and the receiver.

## Study 2

### Research Questions

Study 1 gave us some first evidence that the communication styles adopted by individuals in communicating a displeasing truth are influenced by the individuals’ attachment styles. However, from this first study, it was not clear if people with different attachment styles feel different kinds of emotions about communicating a displeasing truth to another or if they attribute different emotions to their interlocutors. Furthermore, other questions were left unsolved: How do the receivers take the displeasing truth? What are the emotions they feel, and are these emotions in some way affected by their attachment style?

To go in-depth on these issues, we performed a second study to investigate:

1. The effect of the propensity for the different attachment styles on distinct ways of communicating a displeasing truth;
2. The effect of the spokespersons’ attachment styles on the emotions felt and those attributed to their interlocutor.

Furthermore, while the first study only focused on the participant communicating the displeasing truth (spokesperson), this second study also considered the participant who received the displeasing truth (receiver). Therefore, we further investigated:

3. The effect of the receivers’ attachment styles on the emotions they felt and those they attributed to the spokespersons.

In order to investigate these aspects, the experimental procedure was slightly modified.

### Experimental Design

Like in the first study, the independent variables were the spokesperson’s and receiver’s propensity for attachment, namely secure, avoidant, or anxious. However, in the second study, more dependent variables were considered, namely:

A. The way in which the spokesperson communicated the low score to the receiver;
B. The spokesperson’s reactions, namely:
  a. Emotions about his/her high score and the receiver’s low score;
  b. Emotions attributed to the receiver;
  c. Emotions the spokesperson would have felt if he/she had been in the place of the receiver;
  d. How the spokesperson perceived the receiver during the communication of the displeasing truth;
C. The receiver’s reactions, namely:
  a. Emotions about his/her own low score and the spokesperson’s high score;
  b. Emotions he/she would have felt if he/she had been in the place of the spokesperson;
  c. How the receiver perceived the spokesperson during the communication of the displeasing truth.

### Materials and Methods

#### Procedure

This study used the same procedure described for Study 1, but in order to investigate the multiple dependent variables above, the experimental procedure was slightly modified. Two new questionnaires were introduced. Like in the procedure already used in Study 1, the participants (previously unknown to each other) were divided to form pairs. Each participant individually played a bogus computer game, ostensibly to test his/her previous abilities. Subsequently, one participant (the spokesperson) was randomly given the task to communicate to the other one (the receiver) the bogus low score obtained in the computer game (displeasing truth). Three minutes was given to the pair to decide whether to participate in the game together with the other pairs, so the spokesperson was put in the position of communicating the very low score to the other participant. Differently from Study 1, after this confrontation between spokesperson and receiver, a questionnaire was administered to both, presenting a list of six emotions: disappointed, proud, embarrassed, guilty, satisfied, and surprised. The participants were asked to rate (on a six-point scale) the extent to which they had felt each emotion when coming to know their score and (again on a six-point scale) the extent to which they had felt the same six emotions when knowing the score obtained by their partner. After completing the questionnaire, the participants were reminded of the appointment for the second part of the procedure.

Similar to the first study, also during the second phase of the procedure of Study 2, all participants completed the ASQ. Before the ASQ, however, the participants of Study 2 were administered a new questionnaire. The questionnaire first reminded them that the average score obtained in their performance of the computer game was 6.2 (on a scale of 0–10). It then asked the participants to indicate whether their own score was higher or lower than the average and if the score obtained by their partner was higher or lower than the average (control questions on the participants’ scores). Like in the first study, the spokespersons had a high score, while the receivers had a very low score – the received displeasing truth. After this task, a list of 11 emotions was presented: angry, disappointed, sorry, happy, proud, embarrassed, guilty, in distress, worried, quiet, and sad. The participants were asked to rate (on a six-point scale) the extent to which they would have felt each emotion if they were in the place of the other participant. Finally, a list of 25 adjectives was presented: uncomfortable, welcoming, friendly, angry, cooperative, disappointed, sorry, happy, proud, cold, embarrassed, awkward, guilty, in distress, encouraging, irritated, confused, worried, reassuring, blunt, strict, safe, surprised, quiet, and sad. Here, the participants were asked to rate (on a six-point scale) the extent to which they attributed each adjective to the other participant during the face-to-face confrontation. Finally, after administering the questionnaires, the researcher debriefed the participants.

#### Participants

Forty-five pairs of undergraduate students participated in the study. While the first study only focused on the participants who had played the role of spokespersons, this second study also considered their partners, the receivers. Therefore, the results of this study refer to 45 spokespersons and 44 receivers (1 receiver did not participate in the second phase of the procedure). All in all, there were 89 participants (59 women and 30 men, mean age 20.6).

### Qualitative Analysis of the Spokespersons’ Communication

In the first study, a dichotomous classification of the communication styles of the spokespersons (the straight or mitigated communication style) was performed. In this second study, our analysis allowed us to provide a finer distinction.

The transcription of the interactions within the 45 pairs results in a corpus of 11,058 words, a total of 135 min. To elaborate a set of categories for the analysis, first an informal overview was performed of the whole corpus, from which some recurrent communication strategies of the spokespersons emerged. When these categories were found to be exhaustive, 226 utterances in the corpus were classified in terms of them by two judges independently, achieving a good level of agreement (*k* = 0.89).

As compared to what was found in Study 1, results from the more in-depth qualitative analysis conducted in Study 2 allowed us to find three main macro-categories of communicative strategies used when conveying a displeasing truth: reticence, mitigation, and frankness. Moreover, each macro-category showed sub-categories, allowing us to catch nuances of the communication of an unpleasant truth that added complexity to the simpler description of Study 1.

#### Reticence

The spokespersons who choose a reticent communication style try to avoid communicating the displeasing truth to the other participant. There are two signals of reticence:

*Delegation*: While talking to the receiver, the spokesperson attempts to delegate the communication of the displeasing truth to the researcher (“*Did the researcher tell you your score?*”);
*Doubt*: The spokesperson shows doubts about his/her role and the task assigned by the researcher of communicating the score to the receiver (“*I don’t know if I can tell you the results.*”).

Both these signals represent the spokesperson’s attempt to evade the task of communicating the displeasing truth to the other participant.

#### Mitigation

For the mitigated communication style, we observed five types:

*Similarity*: The spokesperson claims his/her similarity with the receiver (“*even I didn’t complete the game*”);
*Minimization*: The spokesperson attenuates the importance of the game (“*the scores are irrelevant*”);
*Solidarity*: The spokesperson shows awareness that he/she is communicating a displeasing truth to the receiver (“*unfortunately, you got 3*”);
*Uncertainty*: The spokesperson shows uncertainty about the information he/she is communicating to the receiver (“*you got 3, I think*”), or he/she expresses disbelief at the researcher’s words (“*I don’t believe what the researcher told me*”);
*Lie*: The receiver asks the spokesperson if he/she performed the game incorrectly, and the spokesperson denies (“*Noooo*…*the researcher told me two results that I don’t think are true*”).

These strategies are aimed to mitigate the potentially displeasing impact of the news.

#### Frankness

Within the frank communication style, we observed four types:

*Receiver’s fault*: The spokesperson assigns the responsibility for the minimal chance of winning in the playful competition with the other pairs to the receiver (“*We are way behind because you got a low score.*”);
*Game simplicity*: The spokesperson shows surprise and confusion about the receiver’s low score. By doing so, he/she risks reinforcing the receiver’s self-attribution of failure (“*It’s impossible to fail in this game.*”);
*Own skill*: The spokesperson emphasizes one’s own skill in the game and thus risks underlining the difference between own score and the receiver’s (“*I didn’t know that the time to play was limited, but still I was fast.*”);
*Extreme frankness*: The spokesperson communicates with very frank expressions, often combined with laughter (“*You sucked.*”).

The signals of frankness, as opposed to those of mitigation, might even amplify the potentially displeasing impact of the news. Our hypothesis was that the spokespersons who choose these extremely frank expressions have difficulty imagining the possible discomfort of the receivers about their own low score, which was both lower than the average and lower than the score obtained by the spokesperson. For each spokesperson, the frequencies of each communicative strategy (reticence, mitigation, or frankness) were calculated.

### Hypotheses

Based on the working model of secure, avoidant, and anxious attachment, as well as on the analysis of the spokesperson’s three possible communication styles, frank, mitigated, and reticent, the following predictions were made:

1. Both spokespersons with secure and avoidant attachment would choose frank communication;
2. Spokespersons with anxious attachment would choose mitigated and reticent communication.

Regarding the spokesperson’s and the receiver’s reactions, we predicted that the participant’s self-assessed emotions and perceptions would be consistent with the characteristics of the internal working model of each attachment style:

1. For individuals with secure attachment, emotions typical of *positive self-image* (the self as worthy of love and support), such as pride and satisfaction, and reactions linked to *positive image of the others* (other people seen as trustworthy and available), such as perception of the other as friendly;
2. For those with anxious attachment, emotions and perception linked to a *negative self-image and high dependency* (a positive self-regard requires external validation or can only be maintained by others’ ongoing acceptance), e.g., disappointment at one’s high score and guilt or embarrassment for the receiver’s low score, and perception of the other as angry and cold;
3. For those with avoidant attachment, reactions stemming from *low dependency and high avoidance of intimacy* (people avoid close contact with others as a result of their expectations of aversive consequences), e.g., pride and satisfaction about their high score, and perception of the receiver as uncooperative and unwelcoming.

### Quantitative Analysis

First, the answers to the control questions about participants’ scores were verified. We found that the spokespersons understood that their score was higher than the average and that the receivers understood that their score was lower than the average. This result confirms the validity of the procedure. Since in Study 2, more participants filled in the ASQ, it was possible to calculate the scores of the propensity for secure, avoidant, or anxious attachment, by performing a factor analysis.

Regarding the “propensity for secure attachment,” the factorial analysis (items 1, 2, 3, 19, 31, 33 reversed, 37, and 38) explained 31.61% of the variance. All of the factor scores were above.42, except item 2, which was removed from the calculations.

Regarding the “propensity for anxious attachment,” the factorial analysis (items 11, 13, 15, 18, 22, 24, 27, 29, 30, 31 reversed, 33, and 38 reversed) explained 35.16% of the variance. All of the factor scores were above.38, except items 11, 29, and 31 reversed.

Regarding the “propensity for avoidant attachment,” the factorial analysis (items 3 reversed, 5, 8, 9, 10, 14, 16, 17, 19 reversed, 20 reversed, 21 reversed, 23, 25, 34, and 37 reversed) explained 25.72% of the variance. All of the factor scores were above.33, except items 8, 9, 10, 23, 25, and 37 reversed.

Furthermore, Cronbach’s alpha was measured for each attachment. The values were 0.83 for the propensity for anxious attachment, 0.77 for the propensity for avoidant attachment, and 0.62 for the propensity for secure attachment.

To explore the effect of the spokesperson’s propensity toward secure, anxious, or avoidant attachment on his/her communication style, his/her emotions and perceptions, and the effect of the receiver’s propensity for secure, anxious, or avoidant attachment on his/her emotions and perceptions, the participants were distinguished into high and low compared to the average of the items for each propensity for attachment (3.70 for the propensity for secure attachment; 3.20 for the propensity for anxious attachment; and 3.87 for the propensity for avoidant attachment). Using this method, we obtained three independent variables for the spokesperson and three independent variables for the receiver.

### Results

This section overviews the results of Study 2, first with regard to the dependent variables connected to the spokesperson, followed by those concerning the receiver.

### Results Concerning the Spokespersons

#### The Spokesperson’s Communication Style

**FIGURE 1:**
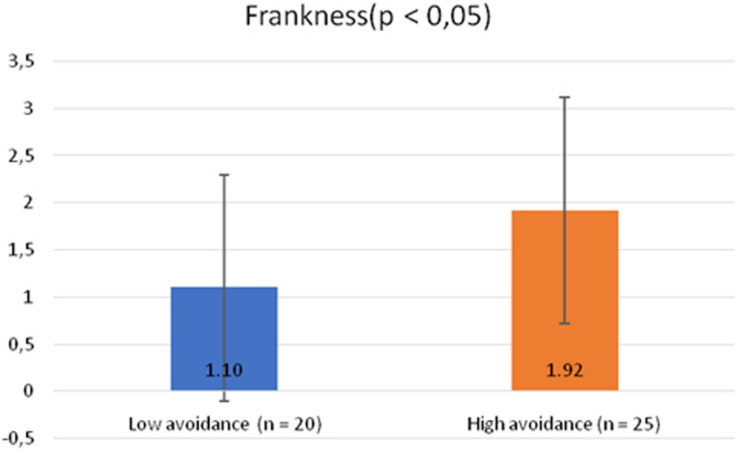
The effect of the spokesperson’s avoidant attachment (high vs. low) on his/her communication style (reticence, frankness, or mitigation).

To explore the effect on the communication style (reticence, frankness, or mitigation) of the spokespersons with high vs. low secure, anxious, and avoidant attachment, we performed analyses of variance (ANOVAs). The first two analyses did not produce significant results. On the contrary, the third ANOVA showed that high avoidant spokespersons [mean (*M*) = 1.92; standard deviation (*SD*) = 1.44] chose franker communication [*F*(1, 44) = 3.93; *p* < 0.05; *n*^2^ = 0.084] compared to low avoidant ones (*M* = 1.10; *SD* = 1.20) (Figure 1).

We performed an ANOVA for each of the effects investigated.

#### The Spokesperson’s Emotions About His/Her Own High Score

The ANOVA exploring the effect of the spokesperson’s propensity for anxious attachment (high/low) on the emotions felt about his/her high score showed that the highly anxious spokespersons (*M* = 0.46; *SD* = 0.83) felt more embarrassed about their high score [*F*(1, 44) = 3.57; *p* < 0.065; *n*^2^ = 0.077] than low anxious ones (*M* = 0.10; *SD* = 0.3).

#### The Spokesperson’s Emotions If He/She Had Been in the Place of the Receiver

Concerning the effect of the spokesperson’s anxious attachment on the emotions he/she would have felt if he/she had been in the place of the receiver, the ANOVA showed that the spokespersons with high anxious attachment would have felt sadder [*F*(1, 44) = 4.64; *p* < 0.03; *n*^2^ = 0.10] and more in distress [*F*(1, 44) = 5.95; *p* < 0.019; *n*^2^ = 0.12] compared to the low anxious ones (Table 1).

**TABLE 1:** The effects of anxious and secure attachment on the spokesperson’s imagined emotions if he/she had been in the place of the receiver.

Emotions	Propensity for *anxious* attachment	Means	*SD*
	High	1.73	1.45
Sad	Low	0.9	0.99
	High	2	1.51
In distress	Low	1	1.14

**Emotions**	**Propensity for *secure* attachment**	**Means**	***SD***

In distress	High	1.05	1.35
	Low	1.95	1.36

*Means, mean of emotions; SD, standard deviation.*

The spokespersons with high secure attachment (*M* = 1.05; *SD* = 1.35) would have felt less in distress if they had been in the place of the receiver [*F*(1, 42) = 4.77; *p* < 0.035; *n*^2^ = 0.10] compared to the low secure spokespersons (*M* = 1.95; *SD* = 1.36) (Table 1).

Furthermore, in the spokespersons with a high propensity for avoidant attachment, there was a positive Pearson’s correlation coefficient between the worry that the spokesperson would have felt, if he/she had been in the place of the receiver, and the reticent communication style (*r*^2^ = 0.44; *p* < 0.031). Therefore, the highly avoidant spokespersons who chose a reticent communication style would have felt more worried if they had been in the place of the receiver.

#### The Spokesperson’s Perception of the Receiver

The ANOVA that explored the effect of the spokespersons’ attachment on their perception of the receiver showed that the high secure spokespersons (*M* = 0.63; *SD* = 1.01) perceived the receiver as less worried [*F*(1, 40) = 4.02; *p* < 0.05; *n*^2^ = 0.09] during the displeasing communication compared to the low secure ones (*M* = 1.41; *SD* = 1.4) (Table 2).

**TABLE 2:** The effects of the spokesperson’s secure and avoidant attachment on his/her perception of the receiver.

Perception	Propensity for *secure* attachment	Means	*SD*
	High	0.63	1.01
*Worried*	Low	1.41	1.4

**Perception**	**Propensity for *avoidant* attachment**	**Means**	***SD***

*Collaborative*	High	4.5	0.74
	Low	3.68	1.2

*Means, mean of perception.*

Again, the spokespersons with a high propensity for avoidant attachment (*M* = 4.5; *SD* = 0.74) perceived the receiver as more collaborative [*F*(1, 40) = 7.03; *p* < 0.01; *n*^2^ = 0.15] during the displeasing communication compared to the low avoidant spokespersons (*M* = 3.68; *SD* = 1.2) (Table 2).

Furthermore, there was a significant Pearson’s correlation coefficient between the spokespersons’ propensity for avoidant attachment, their emotions about the low score of the receiver, and their communication style. This finding indicates that the high avoidant spokespersons who choose franker communication feel more surprised about the low score of the receiver (*r*^2^ = 0.45; *p* < 0.020).

### Results Concerning the Receivers

The results below refer to the participants who received the displeasing truth: their score in the computer game was lower than the average.

#### The Receiver’s Emotions About His/Her Low Score

The ANOVA that explored the effect of the receivers’ attachment on their emotions about their low score showed that the receivers with high anxious attachment felt less satisfied [*F*(1, 42) = 11.79; *p* < 0.001; *n*^2^ = 0.22] and more embarrassed [*F*(1, 42) = 8.11; *p* < 0.007; *n*^2^ = 0.16] about their low score compared to the low anxious receivers (Table 3).

**TABLE 3:** The effects of anxious and secure attachment on the receiver’s emotions about his/her low score.

Emotions	Propensity for *anxious* attachment	Means	*SD*
	High	0.63	0.88
Satisfied	Low	1.78	1.15
	High	2.06	1.52
Embarrassed	Low	0.81	1.30

**Emotions**	**Propensity for *secure* attachment**	**Means**	***SD***

	High	1.64	1.19
Satisfied	Low	0.8	1.01

*Means, mean of emotions.*

On the contrary, the ANOVA showed that the high secure receivers (*M* = 1.64; *SD* = 1.19) felt more satisfied [*F*(1, 42) = 5.38; *p* < 0.02; *n*^2^ = 0.11] about their low score compared to the low secure receivers (*M* = 0.8; *SD* = 1.01) (Table 3).

#### The Receiver’s Emotions About the High Score of the Spokesperson

The receivers with high anxious attachment felt more embarrassed [*F*(1, 40) = 20.10; *p* < 0.001; *n*^2^ = 0.34], less satisfied [*F*(1, 40) = 0.76; *p* < 0.03; *n*^2^ = 0.10], more guilty [*F*(1, 40) = 4.98; *p* < 0.03; *n*^2^ = 0.11], and more disappointed [*F*(1, 40) = 7.09; *p* < 0.01; *n*^2^ = 0.15] about the high score of the spokesperson compared to the receivers with low anxious attachment (Table 4).

**TABLE 4:** The effect of the receiver’s anxious attachment on his/her emotions about the high score of the spokesperson.

Emotions	Propensity for *anxious* attachment	Means	*SD*
	High	2.47	1.99
Embarrassed	Low	0.46	0.85
	High	2.27	1.83
Satisfied	Low	3.35	1.32
	High	1.60	1.76
Guilty	Low	0.58	1.17
	High	1.27	1.53
Disappointed	Low	0.27	0.87

*Means, mean of emotions.*

#### The Receiver’s Perception of the Spokesperson

The receivers with a high propensity for anxious attachment perceived the spokesperson as more uncomfortable [*F*(1, 39) = 6.30; *p* < 0.01; *n*^2^ = 0.14], less happy [*F*(1, 39) = 6.06; *p* < 0.01; *n*^2^ = 0.13], more embarrassed [*F*(1, 39) = 10.96; *p* < 0.002; *n*^2^ = 0.22], more in distress [*F*(1, 39) = 11.68; *p* < 0.002; *n*^2^ = 0.23], and less safe [*F*(1, 39) = 4.74; *p* < 0.03; n^2^ = 0.11] during the displeasing communication compared to the receivers with low propensity for anxious attachment (Table 5).

**TABLE 5:** The effect of the receiver’s anxious attachment on his/her perception of the spokesperson.

Perception	Propensity for anxious attachment	Means	*SD*
	High	1.53	1.24
Uncomfortable	Low	0.68	0.9
	High	1.53	1.45
Happy	Low	2.72	1.48
	High	2.07	1.58
Embarrassed	Low	0.76	0.92
	High	2.07	1.3
In distress	Low	0.8	1.00
	High	2.07	1.10
Safe	Low	2.96	1.30

*Means, mean of perception.*

#### The Receiver’s Imagined Emotions If He/She Had Been in the Place of the Spokesperson

The receivers with high anxious attachment (*M* = 1.6; *SD* = 1.63) would have felt more in distress [*F*(1, 39) = 10.77; *p* < 0.002; *n*^2^ = 0.22] in the place of the spokesperson compared to the low anxious receivers (*M* = 0.4; *SD* = 0.64) (Table 6). On the contrary, the receivers with high secure attachment would have felt more quiet [*F*(1, 39) = 5.52; *p* < 0.02; *n*^2^ = 0.12], more proud [*F*(1, 39) = 4.60; *p* < 0.03; *n*^2^ = 0.10], and more happy [*F*(1, 39) = 4.26; *p* < 0.04; *n*^2^ = 0.10] if they had been in the place of the spokesperson (Table 6).

**TABLE 6:** The effects of the receiver’s anxious and secure attachment on the imagined emotions if being in the place of the spokesperson.

Emotions	Propensity for *secure* attachment	Means	*SD*
	High	3.85	1.22
Quiet	Low	2.71	1.81
	High	3.42	1.41
Proud	Low	2.29	1.89
	High	3.27	1.53
Happy	Low	2.14	1.83

**Emotions**	**Propensity for *anxious* attachment**	**Means**	***SD***

	High	1.6	1.63
In distress	Low	0.4	0.64

*Means, mean of emotions.*

### Discussion

This second study allowed us to explore in greater depth how the strategies to communicate a displeasing truth are linked to attachment styles of the spokesperson and to explore receivers’ reactions as well.

First of all, a more complex and nuanced description of communication strategies was found. Together with mitigation, already found in Study 1, reticence emerged as a way to cope with the negative aspects of conveying the displeasing truth. Frankness too showed not only a positive facet, linked to a collaborative attitude to present the receiver with the plain truth, but also a negative aspect of extreme frankness that disregarded receivers’ feelings. Different attachment styles seemed not directly linked to the choice of a specific communication strategy, if not for the spokespersons with high avoidant attachment, who showed a tendency to choose a franker and somehow brutal communication. This result is consistent with the negative model of the other (Bartholomew and Horowitz, 1991), which characterizes avoidant attachment: a lack of trust in the other, fear of intimacy, and avoidance of closeness due to expectations that others will not be available and supportive (Brennan et al., 1998; Cole, 2001). Therefore, avoidant spokespersons might choose a franker communication style because they do not care about the possible negative consequences that revealing an unpleasant truth might have on a relationship. Coherently with theoretical frameworks assuming that individuals with avoidant attachment choose strategies to increase their autonomy and distance in the relationships (Cassidy and Kobak, 1988), such a frank communication style might serve as a strategy used by avoidant individuals to keep everyone out, sending signals that discourage the search for emotional closeness in others.

Being contemptuous of others – a characteristic of individuals with avoidant attachment – might also explain why spokespersons with high avoidant attachment who chose a frank communication style felt more surprised about the low score of the receiver. Probably they thought the game was easy to play, and their surprise might include a negative evaluation of the receiver’s performance. This might support the interpretation that the avoidant spokespersons choose a frank communication style as an indication of their low evaluation of the receiver and a means to keep a distance from others. Apart from this direct link of the avoidant attachment to franker communication of the displeasing truth, all other effects of attachment styles shown in the study are linked to the perceptions and emotions felt by both spokespersons and receivers during this difficult communication. More precisely, we may consider these effects in accordance with the different attachment styles of both spokespersons and receivers. In fact, being involved in the communication of a displeasing truth is a difficult personal and social condition that could activate the attachment system of the spokesperson and of the receiver and mold their perceptions and emotions during this challenging interaction according to their own specific internal working models.

The results of Study 2, which not only observed actual communications of the spokesperson but also explored perceptions and emotions of both spokespersons and receivers, can therefore be grouped according to their attachment styles.

#### Perceptions and Emotions of Avoidant Spokespersons

Spokespersons with high avoidant attachment perceive the receiver as more collaborative during the unpleasant communication compared to those with low avoidant attachment. One can argue that the highly avoidant spokespersons have difficulty imagining the possible discomfort of the receivers about their low score. However, the highly avoidant spokespersons who chose a reticent communication style would have felt more worried if they had been in the place of the receiver. We can assume that the highly avoidant spokespersons who chose a reticent rather than a frank communication style were able to empathize with the receivers and imagine the potentially unpleasant impact of the displeasing truth. Nevertheless, worry being the emotion attributed by those spokespersons to receivers, one can wonder if this emotion could be linked to the implicit meaning of personal failure attributed by these spokespersons to the low score of receivers. Therefore, this empathic attitude could be seen as a benevolent facet of the more general negative model of the other that characterizes this attachment style.

#### Perceptions and Emotions of Anxious Spokespersons

In line with the negative model of self (Bartholomew and Horowitz, 1991) that characterizes anxious attachment, the spokespersons with high anxious attachment would have felt sadder and more in distress, compared to those with low anxious attachment, if they had been in the place of the receiver. The working model of anxious attachment includes preoccupation with intimacy, jealousy, and fear of abandonment, as well as a dependency on close others’ approval rather than an internal sense of self-worth (Brennan et al., 1998; Cole, 2001). Since the individuals with anxious attachment have low self-esteem and feel unworthy of love, we might assume that spokespersons with this attachment style would have felt sadder and more in distress if they had obtained a low score because it would have further undermined their image and self-esteem. At the same time, we hypothesize that the characteristics of individuals with anxious attachment might enable them to imagine the possible discomfort of the other.

Regarding the spokesperson’s emotions, those with high anxious attachment felt more embarrassed about their high score. This result seems to support the interpretative hypothesis that the individuals with anxious attachment can understand that the difference between the two scores might have an unpleasant impact on the receiver. Since their working model includes preoccupation with jealousy, perhaps this embarrassment could be linked to the fear of negative reactions of the other, due not only to empathic concerns but also to a fear of the social comparison implicit in the truthful communication of both scores that the spokespersons are asked to convey to their less successful partners.

#### Perceptions and Emotions of Secure Spokespersons

The spokespersons with high secure attachment would have felt less distress compared to those with low secure attachment if they had been in the place of the receiver. Given that individuals with secure attachment have high self-esteem and are not afraid of being rejected by others (Mikulincer and Shaver, 2016), we may think that spokespersons with such an attachment style would have felt less in distress if they had obtained a low score because it would not have undermined their high image and self-esteem. Another result consistent with the influence of an internal working model of secure attachment during the communication of a displeasing truth is that the spokespersons with a high propensity for secure attachment perceived the receiver as less worried during this unpleasant communication compared to those with low secure attachment. Individuals with secure attachment feel worthy of love and are not afraid to lose the relationship with the other, and thus, the highly secure spokespersons probably perceived the receiver as less worried because they were not afraid that the difference between the two scores would have negative effects on their relationship with the receiver, as it happened in the case of spokespersons with anxious attachment.

Together with these effects on perceptions and emotions of the spokespersons, Study 2 allowed us to grasp how the internal working model linked to their attachment style also influenced perceptions and emotions of receivers of a displeasing truth.

#### Perceptions and Emotions of Anxious Receivers

First, the receivers with high anxious attachment felt less satisfied and more embarrassed about their low score compared to those with low anxious attachment. This result seems to confirm the low self-esteem in individuals with anxious attachment (Mikulincer and Shaver, 2016). The lack of self-confidence might explain the receivers’ dissatisfaction and embarrassment about their low scores.

Another interesting result is that the receivers with high anxious attachment felt more embarrassed, less satisfied, more guilty, and more disappointed about the high score of the spokesperson compared to those with low anxious attachment. Consistent with the working model of anxious attachment, we hypothesize that this is so because their scores were lower than the average and, therefore, they might fear penalizing their partner in a game with the other couples.

Another result that seems to confirm the internal working model of anxious attachment is that the receivers with high anxious attachment perceived the spokesperson as more uncomfortable, less happy, more embarrassed, more in distress, and less safe compared to those with low anxious attachment. We hypothesize that the receivers with high anxious attachment have imagined being a spokesperson, and because the individuals with anxious attachment are afraid of being abandoned and need the other’s approval, they might fear the potential consequences of unpleasant communication on their relationship with others and on their image. Further, they felt those emotions because they would have felt them had they been in the place of the spokesperson. This interpretative hypothesis can also explain the result that the receivers with high anxious attachment would feel more in distress if they had been in the place of the spokesperson compared to the receivers with low anxious attachment.

#### Perceptions and Emotions of Secure Receivers

The receivers with high secure attachment felt more satisfied about their low score compared to those with low secure attachment. In fact, in contrast to the individuals with anxious attachment, those with secure attachment have high self-esteem, and a low score in a computer game is not sufficient to question the self-esteem of these receivers.

Moreover, in line with the working model of secure attachment, the receivers with high secure attachment would feel quieter, prouder, and happier if they had been in the place of the spokesperson compared to those with low secure attachment. We hypothesize that the receivers with a high secure attachment would feel proud and happy about their high score, if they had been in the place of the spokesperson, because in that case, they would be reaching positive goals, like in a vicarious experience of success (Poggi and D’Errico, 2011). In addition, they would have quietly communicated the unpleasant news to their partner without fear of provoking the jealousy of the other and losing the relationship.

Of course, explaining all these results related to perceptions and emotions of both spokespersons and receivers requires more than referring to their internal working models of attachment styles. It is necessary to consider the ability of an individual to imagine the possible reactions of the other and his/her tendency to attribute his/her own emotions to the other. However, it is interesting to note how expectancies foreseen by attachment theory may contribute to explaining and interpreting these results. Moreover, these data also show that the procedure set in place during the study was able to catch specific nuances of these theoretical expectancies, elicited by the specific personal and social challenge of telling a displeasing truth to another person, a stranger to the spokesperson. Not being based on prior interpersonal knowledge, in fact, the perceptions and emotions of both spokespersons and receivers are mostly built up in reference to their own internal working models, and therefore shed a particular light on reactions to this very specific social situation, which goes even beyond the general expectancies included in the original theoretical model.

## General Discussion

Our first study confirmed the relationship between attachment style and the communication of a displeasing truth. The results of Study 1 show that individuals with a propensity for anxious attachment have more difficulty communicating the displeasing truth; hence, they tend to choose a mitigated communication style to reduce their relational anxiety and modulate the potentially unpleasant impact of the news. In the second study, we applied a more articulated criterion to analyze the verbal content of the spokespersons’ communication: overcoming the previous dichotomous classification – straight vs. mitigated communication style – we found that the spokespersons of our procedure may adopt three different ways (reticence, mitigation, and frankness) of communicating the displeasing truth, and specifically distinguished two types of reticent, five types of mitigated, and four types of frank communication. This more fine-grained analysis allowed us to explore how the spokesperson’s attachment style induced a preference for one of the three. Further, in Study 2, more research issues were tackled: two new questionnaires administered to both spokespersons and receivers explored each participant’s emotions about one’s own score, the partner’s score, the imagined emotions if he/she were in the place of the partner, and his/her perception of the partner. So, the receivers’ reactions were investigated too, allowing us to examine how their attachment style may affect their emotions and perception of the spokesperson. Results of this new analyses show that perceptions and emotions of both spokespersons and receivers of a displeasing truth are influenced by the internal working model linked to their own attachment style. The specific personal and social challenge due to the need of speaking and receiving a difficult truth during an interpersonal communication with a stranger, set in place by the original procedure tested in Study 1 and developed in greater depth in Study 2, makes it evident how perceptions and emotional relations elicited during this communication may be influenced by the internal working models of all partners of this communication, even beyond the expectancies formulated by the theoretical model describing the consequences generally expected by the attachment theory.

## Conclusion

This paper has explored the underinvestigated issue of how communication of a displeasing truth can be influenced by the attachment style of both senders and receivers of this difficult communication. The complexity of the adopted procedure, along with the time-consuming analysis of the verbal data, necessarily restricted the participants’ sample of the two studies presented, but the results of this first exploration are encouraging. In the first explorative study, we observed that the communicative strategies used by the spokesperson to convey to the receiver his/her poor score in a game was influenced by the spokesperson’s attachment style, as assessed by the ASQ by Feeney et al. (1994). The results generated by our procedure showed that anxious participants more frequently chose a mitigated communication strategy when conveying a displeasing truth. Nevertheless, this study had some limitations. First, due to the low number of participants, the ASQ scores could not be fully elaborated. Second, the observation was focused only on the spokesperson. Thus, a second study was planned in which, by observing a higher number of participants, we could differentiate a high vs. low similarity of each participant to scores that distinguished each attachment style, as assessed by the ASQ. Moreover, a more in-depth qualitative analysis of the verbal utterances of spokespersons was performed, leading to differentiation of the strategies used by the spokesperson into reticence, mitigation, and frankness, this last definition comprising either a clear communication or even a more brutal communication of the displeasing truth. Finally, Study 2 also investigated the perceptions and emotions of both spokespersons and receivers in this difficult interpersonal communication, in order to explore their links, if any, to their own specific attachment style (secure, anxious, or avoidant).

The results of this second study show that spokespersons with a high propensity for avoidant attachment chose franker communication when conveying to the receivers their poor scores compared to spokespersons with a low propensity for avoidant attachment. In accordance with more general assumptions of avoidant attachment (Bartholomew and Horowitz, 1991; Brennan et al., 1998; Cole, 2001), these data suggest that avoidant spokespersons may use a brutally frank communication truth as a “de-activating” strategy (Cassidy and Kobak, 1988), in order to maximize their relational distance from receivers.

Also, results on perceptions and emotions of both spokespersons and receivers show interesting nuances of the influence of the attachment styles on this challenging interpersonal communication. Taken together, these results suggest that the internal working models linked to specific attachment styles influenced the emotions and social impressions of both members of the couple of participants who, strangers to one another, were asked to be involved in a difficult interpersonal communication. The specific procedure set in place in fact elicited a social comparison between the two, whereby one who was put in a better social position had to communicate bad news about the other’s poor performance. The results of Study 2 suggest that each participant’s internal working models were used to cope with the difficult communication, whether in the role of spokesperson or receiver. A further suggestion emerging from our data is that dimensional measures of attachment propensity can help to develop more complex explanations, in psychological terms, of a given behavior, rather than simply find a correspondence between a prototypical profile and its associated behavior.

More generally speaking, to understand how people cope with the personal and social challenge of communicating or receiving a displeasing truth, one should reference the internal working models of participants as well as their capacity to foresee the uneasiness felt by the other person (Axia, 1999) and the pragmatic consequences of the inconvenient truth on the social and personal face of the receiver (Goffman, 1967; Brown and Levinson, 1978); and within the literature on politeness, specifically the studies that show how pragmatic consequences can be appreciated if communication is clear and nice. While the first dimension is well explained by the theory of Grice (1975), which indicates that clear communication maximizes the informative contents, a speaker can be defined as nice when he/she masters the social processes between communicative actors (Lakoff, 1973; Leech, 1983). In other words, if a speaker is clear by avoiding any misunderstanding, he/she is also nice when he/she is aware of the other person’s need to protect his/her face. Our studies highlight that participants with a high propensity toward avoidant attachment choose to be extremely frank when communicating a displeasing truth to receivers, with the aim of being clear while neglecting being nice. Participants with a high propensity toward anxious attachment, on the contrary, while fulfilling the aim to be nice, are at the same time less frank, mitigating the clear communication of the displeasing truth. Also, the perceptions and emotions of receivers seem to look for a difficult balance between understanding inconvenient information and protecting the interpersonal relation with the spokesperson. While internal working models linked to secure attachment seem to enable both spokespersons and receivers not to worry about the effects of communicating the inconvenient truth, models linked to anxious attachment seem to expose both communication partners to the disruptive consequences of the social comparison implicit in the difference between the high score of the spokesperson and the low score of the receiver.

Together with the promising results on an understudied issue, the studies presented in this article also have some limitations. First of all, the sample of both studies is not balanced for gender, and due to the priority of our need to test the novelty of the original procedure used in the two studies, we did not explore differences linked to gender roles. More research should be done in order to explore whether gender differences arise in similar studies. Another limitation of the two studies is the fact that only verbal contents were analyzed. Future studies might explore the communication of a displeasing truth in more depth. For instance, the emotions of both spokesperson and receiver could be explored by direct observation using tools such as the Facial Action Coding System (Ekman et al., 2002) and not only questionnaires of self-reported emotions. Finally, a better grasp of multiple nuances of the communicative strategy chosen by a spokesperson when communicating an inconvenient truth might be attained by including an analysis of verbal contents in a more comprehensive multimodal analysis (Poggi, 2007), taking into account not only words and facial expressions but also gestures, gaze, postures, and the intertwining of the meanings they all convey in both spokespersons’ and receivers’ communication.

Besides a theoretical advancement of the understanding of the relationships between personality and communication, this work might be also of use on the application side, since the possibility of detecting communicative strategies from different attachments styles, or the other way around, might help to build more sophisticated systems for user modeling and the planning of personalized systems.

## Data Availability Statement

The datasets generated for this study are available on request to the corresponding author.

## Ethics Statement

The manuscript was reviewed and ethically approved by the committee of doctorate’ psychology and cognitive science of the Sapienza University of Rome. The patients/participants provided their written informed consent to participate in this study. The procedure was conducted in accordance with the ethical requirements of research prescribed by AIP (Associazione Italiana di Psicologia, Italian Psychology Association).

## Author Contributions

IS: literature review, method, and qualitative analysis. GL: method, literature review, and writing. FD’E: quantitative analysis, discussion of the results, and writing. IP: qualitative analysis, literature review, and writing.

## Conflict of Interest

The authors declare that the research was conducted in the absence of any commercial or financial relationships that could be construed as a potential conflict of interest.
